# Reversing the Trends toward Shorter Lives and Poorer Health for U.S. Women: A Call for Innovative Interdisciplinary Research

**DOI:** 10.3390/ijerph15091796

**Published:** 2018-08-21

**Authors:** April Schweinhart, Janine Austin Clayton

**Affiliations:** 1 Pacific Institute for Research and Evaluation, Beltsville, MD 20705, USA; aschweinhart@pire.org; 2 National Institutes of Health, Office of Research on Women’s Health, Beltsville, MD 20892, USA

**Keywords:** women’s health, health disparities, sex and gender, interdisciplinary research

## Abstract

The United States (U.S.) is a leader and innovator in biomedicine, yet trails behind for many key health indicators, especially for women. This paper highlights key evidence indicating that not only is the state of women’s health in the U.S. lagging, but it is at risk for falling off the curve. Women’s health care remains fragmented; research in the field can be disconnected and difficult to integrate across disciplines—silos prevail. Structural obstacles contribute to this lack of cohesion, and innovative, interdisciplinary research approaches which integrate the multidimensional aspects of sex and gender, and race and ethnicity, with a life course perspective are sorely needed. Such synergistic, scientific strategies have the potential to reverse the trend towards shorter life expectancy and poorer health for women in the U.S. The National Institute for Health (NIH) seeks to raise the bar for the health of all women by tackling these issues through enhancing the relevance of biomedical research to the health of women and driving the sustained advancement of women in biomedical careers.

## 1. Introduction

**Figure 1:**
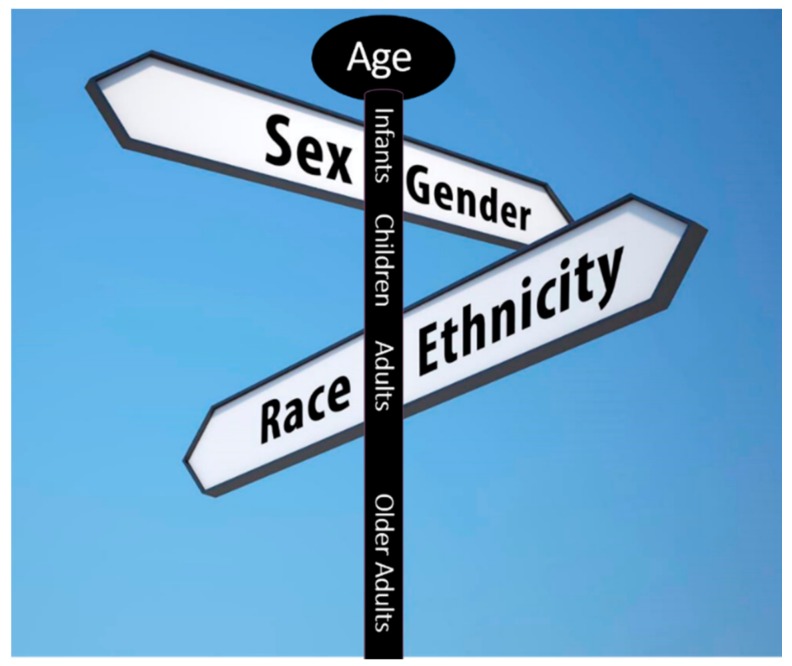
When considering the direction in which to take biomedical research, the influences of sex, gender, race, ethnicity, and the entire life course must all be taken into account in an integrated fashion. The graphic illustrates the intersectional relationships between these essential domains.

Health risks, protective factors, and disease profiles differ for men and women of different races and ethnicities and across the life course [1,2]. Historically, health decisions for women were often based on research done primarily with men. After the adverse effects of thalidomide on pregnant women in the 1960s, women were often excluded from clinical trials and many researchers are still wary of including premenopausal women due to the potential risk of pregnancy and infertility [3,4]. However, without appropriate representation of men and women in clinical research and attention to sex as a biological variable in pre-clinical research, there will be gaps in knowledge and understanding of differences by sex with regard to treatment and response [3]. This paper represents information presented at the 2017 Proceedings of Research Centers at Minority Institutions (RCMI) Translational Science Conference. As such, it is not meant to be a complete review of the literature, but rather, illustrates key points and relevant factors affecting the current state of the health of women. Beginning with a short review of the existing state of research in the United States (U.S.), this paper highlights salient examples of the intersecting influences of gender, race/ethnicity, and age on the health of women, supporting the imperative for a novel, multidimensional approach, encompassing specific attention to the stages of a woman’s life through integrative, interdisciplinary investigation (See Figure 1).

At a federal level, the Department of Health and Human Services (HHS) recognized women’s health as an important issue deserving of special attention in 1984 and created the HHS Office of Women’s Health (OWH) in 1991. With HHS, many other women’s health offices, including at the National Institutes of Health (NIH) and the Food and Drug Administration (FDA), work to address the health needs of women. The NIH Office of Research on Women’s Health (ORWH) is the first office of Public Health Service and the first office at NIH to specifically promote women’s health and research. In 1990, congress mandated the creation of ORWH to advise the NIH on matters related to the health of women, to ensure that research supported by NIH adequately addressed this topic, including strengthening and enhancing research concerning conditions that specifically affect women, to make sure women were appropriately included in NIH-funded clinical research, and to develop opportunities for women in biomedical careers [5]. Since then, the office has made tremendous progress. Today, over half of participants in NIH-supported clinical trials are women, and NIH continues to expand efforts to enhance the inclusion of women, including through partnership with OWH at U.S. FDA to ensure the inclusion of diverse women in clinical trials [6]. ORWH has also created an NIH Inclusion Outreach Toolkit, an online tool to improve the engagement, recruitment, and retention of women in clinical research. The toolkit’s value is in providing successful strategies used to overcome challenges related to recruiting and retaining women from diverse backgrounds and of all ages. The toolkit and each case study’s results can be found on the ORWH website (https://orwh.od.nih.gov/). Yet, gaps in knowledge about women’s health research and sex and gender differences remain, including a dearth of understanding of healthy aging and the complex health dynamics of women across the life course.

The U.S. is a world leader in healthcare spending, biomedical research, and innovation for the diagnosis and treatment of diseases. In the last two years alone, the U.S. has produced astounding innovations in medical treatment and diagnosis, including: Tissue Nanotransfection (TNT), a new technology enabling the generation of tissues within the patient’s own body [7]; prompt detection of nucleic acids with previously unknown sensitivity [8]; rapid and efficient cancer drug development with molecular target technology [9]; and the development and FDA approval of the “artificial pancreas”, an implanted device that automatically monitors and adjusts insulin levels for type 1 diabetes patients as young as 14 years of age [10].The NIH is leading the charge in developing a new initiative for precision medicine with the All of Us research program [11]. The goal of this program is to bring together volunteer research participants to make electronic health record and other data from healthy individuals, including those underrepresented in biomedical research, available to researchers across the globe. The All of Us effort will enable researchers to study environmental, behavioral, and individual impacts on genotypic and phenotypic manifestations of disease, as well as overcome issues affecting traditional research designs, such as statistical power.

**Figure 2:**
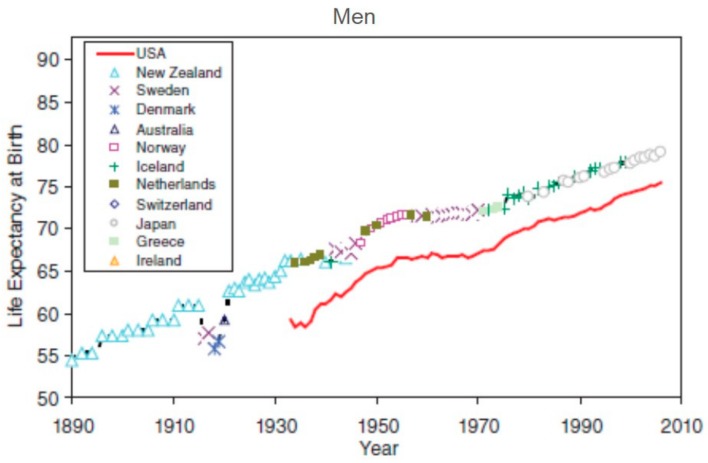

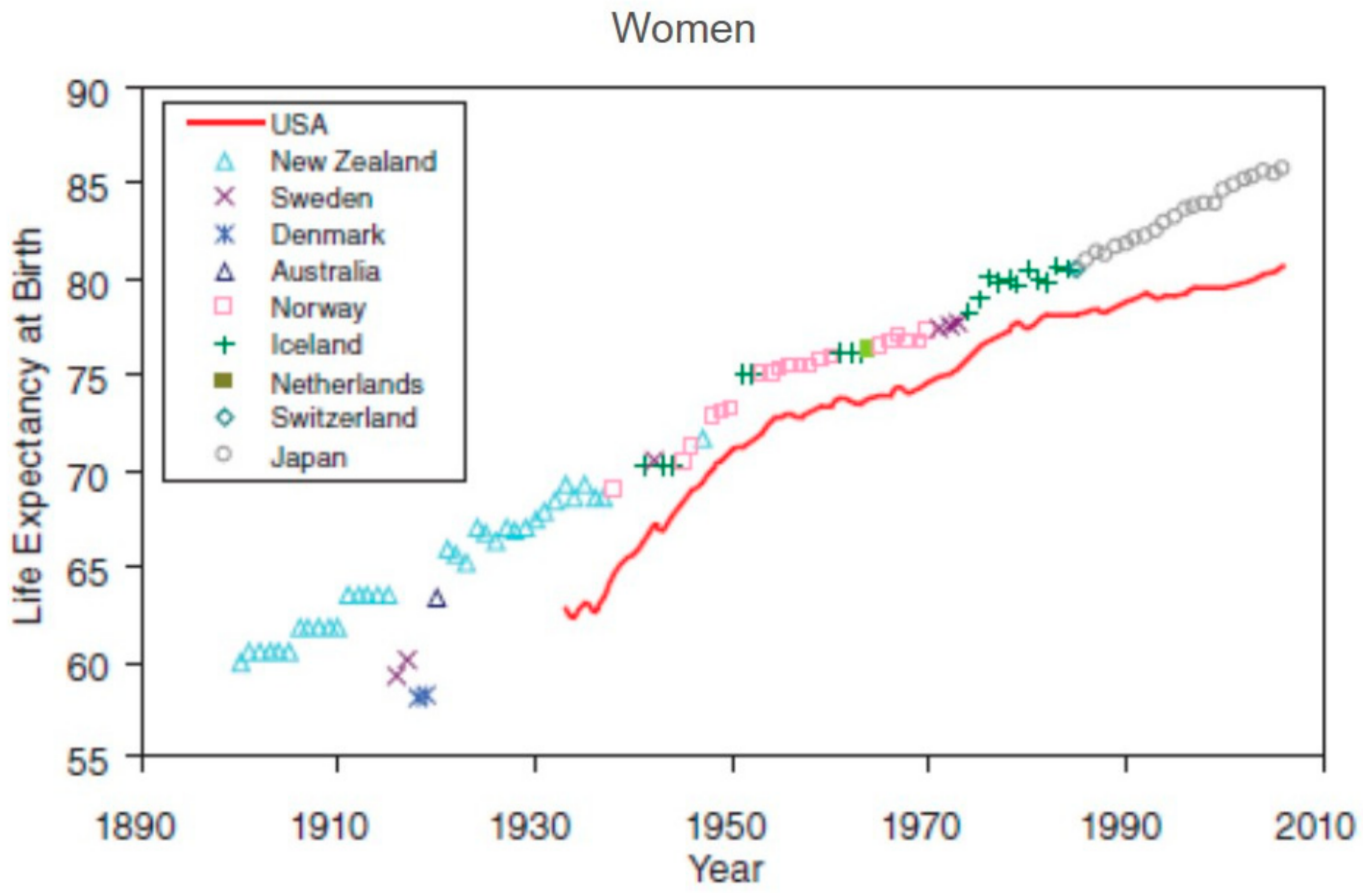
Life expectancy by country and year of origin for men (top) and women (bottom) since 1980 for different industrialized nations (represented by different symbols). U.S. is shown in red. (Reprinted from [12]).

Despite these innovations, the U.S. lags behind similarly economically situated countries in many population health indicators. For example, when comparing life expectancy at birth to other industrialized nations, men and women in the U.S. are well below average. Moreover, while U.S. men’s life expectancy has continued to increase over time, following the trend of other countries, women’s life expectancy has plateaued since 1980 (See Figure 2 [12]). Within the U.S., life expectancy varies dramatically, depending on geography [13]. Looking at women’s mortality across counties in the U.S. shows that, while there have been significant increases in men’s life expectancy since 1985 in 95% of counties, women’s life expectancy has either remained the same or decreased in 45% of counties (over 1400 counties) [14]. At the state-level, Montez and colleagues found that individual-level factors, such as age, race, ethnicity, income, and marital status, can only account for about a third of this variation in mortality rates [15]. Another third was accounted for by state-level factors, such as economic conditions, physical infrastructure, and tobacco environment. To state it differently, the context and environment of women seem to play at least as important of a role in determining their health as individual-level factors. These results illustrate quite clearly that it is necessary to take a multidimensional approach to “raise the bar” for the health of women.

In summary, despite the fact that the U.S. spends more than any other country on healthcare and is a leader in medical innovation, the U.S. ranks nearly last in key health indicators, particularly for women, revealing the need for innovative approaches including a multilevel model of women’s health across the life course. This paper will demonstrate that the health of women is dynamic and requires considering the multifaceted dimensions of her life (i.e., gender, age, race and ethnicity) through interdisciplinary and integrated evaluations using a situated approach. To obtain a comprehensive understanding of changes in women’s health across the life course, it is essential to methodically examine and report health data disaggregated by sex and age as appropriate, to acknowledge and understand differences in women’s health based on race and ethnicity, and analyze longitudinal trends in health data. These key factors—sex and gender, race and ethnicity, and age—form the most important intersection of influences on health and disease (Figure 1). The paper is organized by these three areas and each section will discuss a few key examples to highlight the importance of considering and integrating these factors when it comes to the health of women. With ORWH as the leader, NIH has adopted an interdisciplinary approach that integrates these aspects through biomedical, epidemiological, and life course approaches to fill existing knowledge gaps in women’s health. As opposed to individual or multidisciplinary research, interdisciplinary research integrates information, data, techniques, and tools of two or more disciplines to advance understanding beyond the scope of either single discipline or practice [16,17,18]. The paper concludes by spotlighting NIH and ORWH efforts to fill the gap in women’s health disparities, and to provide a framework for putting science to work for the health of all women.

## 2. Sex and Gender Difference

Women comprise more than 50% of the U.S. population and tend to outlive men. Sex and gender influences and differences in health and disease status between women and men is the rule rather than the exception, and therefore clinicians should expect to see differences in virtually all patients they encounter. With such widespread sex and gender effects, a few major categories which place a heavier burden on women than men have been selected to demonstrate particular patterns of variation: sleep, cardiovascular disease (CVD), diabetes, obesity, and cancer (discussed in the next section) [3]. (For a more complete review of health disparities in reproductive medicine, see Owen et. al., 2013, and for Gender-based approaches to medicine, see Legato and Bilezikian, 2004 [4,19]). One area of health that affects women differently than men is sleep. According to the Center for Disease Control and Prevention, (CDC), adults should get 7 or more hours of sleep per night but, on average, 35.2% of adults ages 18–65 report getting fewer than 7 h [20]. While sleep duration does not differ between men and women, and women report better sleep quality than men, they tend to report more sleep-related complaints, with a higher incidence of insomnia and restless leg syndrome. Sleep disturbances and deficiency are linked to overall health and can affect blood pressure, the risk for obesity, depression, and CVD, the leading cause of death in the U.S. (causing 23.4% of deaths from 1975–2015) [21,22,23]. Heart disease, including myocardial infarction or heart attack, heart failure, coronary heart disease (CHD), stroke, and hypertension, affects 10.7% of the U.S. total population, and as women age, they are disproportionately affected by CVD [24]. Moreover, among all people affected by heart attacks, women have greater fatality rates compared to men [24].

The combination of obesity and diabetes is particularly detrimental for women; women with diabetes have twice the risk of CHD, experience heart attack at earlier ages, and have higher overall mortality rates [25,26]. CHD affects 5.7% of the U.S. population (7.3% men, 4.4% women), hypertension 24.9% (26.2% men, 23.7% women), and stroke 2.8% (equal across sex) [27]. Though CVD may be listed as cause of death, comorbidities are common; obesity and diabetes are risk factors for CVD, and obesity alone is a risk factor for diabetes [28]. As of 2014, 12.6% of adults 20 years of age and older have diabetes and 37.8% are considered obese (having a body mass index (BMI) at or above sex and age specific 85th percentile) and both of these statistics have steadily increased in the past 30 years [23]. Among men and women with diabetes, CVD is the leading cause of death, but there is a higher prevalence of obesity and poorer blood pressure control in women. Diabetes is also a stronger risk factor for stroke in women than men [25,26]. Overall, when it comes to CVD and diabetes, women have poorer cardiovascular outcomes than men.

## 3. Racial and Ethnic Disparities

**Figure 3:**
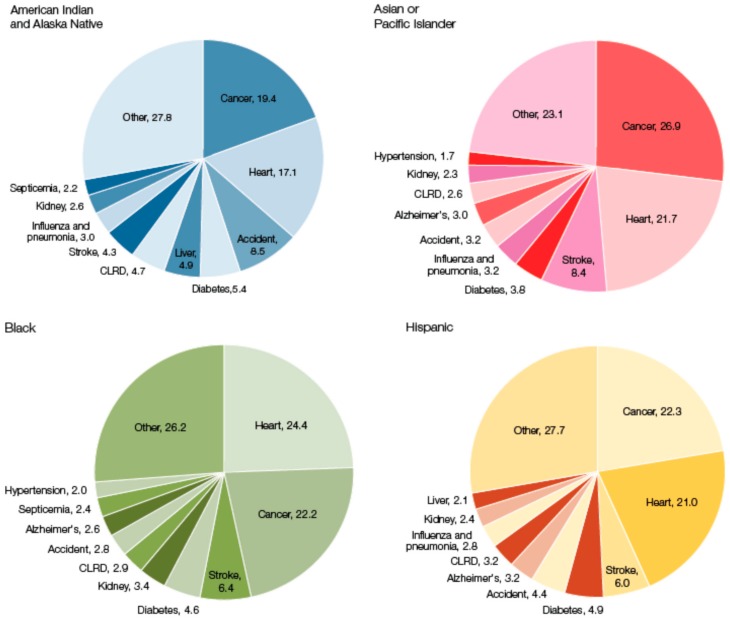
The causes of death for women by race and ethnicity. (Reprinted from the Women of Color Health Data Book [29]).

Just as sex and gender influence health and disease, so too do race and ethnicity. Even when demographic variables are controlled for in analyses of studies which interrogate racial and ethnic differences, disparities persist, implicating biological, genetic, or epigenetic factors [4]. As Figure 3 illustrates, the leading cause of death among women varies across race and ethnicity [29]. For example, for Black and White women, heart disease is the leading cause of death, followed by cancer. In contrast, cancer is the leading cause of death, followed by heart disease, in Asian or Pacific Islander, Hispanic, American Indian, and Alaska Native women [30]. In addition, American Indian and Alaska Native women experience high rates of mortality from unintentional accidents and liver disease, which are less common causes of death in other racial and ethnic groups [30]. Breast cancer is the most common type of cancer among women ages 35–64 and the incidence rate is highest for white women (128.1), followed by Black women (124.3), Alaskan Native or American Indian (91.9) and Hispanic women (91.9), and Asian or Pacific Islander women (out of 100,000) [31]. While the incidence rate is higher for White women, the death rate from breast cancer in African American women exceeds that of white women, and the gap is widening [31]. In fact, one of the most striking racial differences in oncology is the disparity in burden that breast cancer places on African American, as compared to White, women. The disparity is in part due to triple-negative breast cancer (TNBC), an aggressive subtype that disproportionately affects young African American women [32,33]. TNBC is negative for estrogen receptor, progesterone receptor, and ErbB2, a receptor-like tyrosine kinase. Not only are African American women more likely to have TNBC, but they also have a poorer survival rate with TNBC than their White counterparts [32].

Like breast cancer, cervical cancer is another women’s health priority that affects races and ethnicities differently. Fortunately, cervical cancer rates are dropping due to increasing prevention measures, such as Papanicolaou (Pap) screening and the introduction of the human papillomavirus (HPV) vaccine. Cervical cancer affects 1.1% of white women, 1.1% of Black women, 0.7% of American Indian or Alaskan Native women, and 0.3% of Asian women in the U.S. [27]. Across the globe, cervical cancer is the second most common tumor in women, and nearly all cases are caused by about 15 different types of HPV. Prevention of cervical cancer via use of Pap screening, HPV vaccination, and a new carcinogenic HPV DNA test is the key to influencing women’s health worldwide [34]. Unfortunately, women without access to these key medical services have the greatest risk of getting cervical cancer. One factor in contributing to access is health literacy. Asian American women may have higher rates of cervical cancer due to limited understanding of prevention and medical services [35,36]. Among Vietnamese American women, who show lower levels of cervical cancer screening than White women, fewer than two-thirds of women interviewed identified correct beliefs about Pap screening, and over 85% indicated an incorrect opinion that women’s hygiene was associated with cervical cancer [35]. Increasing access to culturally appropriate education and prevention tools for disparately affected populations could decrease the rate of cancer in these populations. Other U.S. minority populations for which cervical cancer and resulting mortality rates remain higher than for White women include American Indians in the Northern Plains, Alaska Natives, and Hispanics along the U.S.-Mexico border. These disparities are likely related to health care access issues [37].

Diabetes mellitus also exhibits inequalities across race and ethnicity; the prevalence of diabetes is higher in Black, Hispanic, American Indian, and Alaska Native women than their White or Asian counterparts. Whereas diabetes affects 8.8% of White individuals and only 8% of Asians, 13.1% of Black or African Americans, 12.2% of Hispanics, 13.1% of Mexican Americans, 14.4% of Native Hawaiian or other Pacific Islanders, and 15.7% of American Indian or Alaskan Natives have diabetes [27]. Diabetes is higher on the list of causes of death for Black and Hispanic women than other racial and ethnic groups as well [30]. This demonstrates the Hispanic paradox; on average, both Hispanic men and women have higher rates of diabetes, obesity, and other risk factors than Whites, making them significantly more disadvantaged in terms of mortality risk [38]. Yet, as a whole, Hispanic men and women tend to live about two years longer than non-Hispanic Whites (across gender). Historically, the Hispanic paradox was explained in one of two ways; either stronger, healthier people are more likely to emigrate, or first-generation immigrants are more likely to return to their home country when they become ill, thus eliminating their death records from U.S. registries [39,40]. More recently, however, Hovarth and colleagues used epigenetic age acceleration of blood samples from multiple racial and ethnic groups and reported lower intrinsic aging rates in Hispanics [41]. These lower aging rates may compensate for the higher mortality risk factors leading to longer lifespans.

Cardiovascular disease is the leading cause of death in the U.S., but also demonstrates differential racial and ethnic impacts. As evidenced in Figure 3, CVD causes more deaths among Black women and fewer deaths among American Indian and Alaska Native women. Overall, in the U.S., Blacks show the greatest mean heart age, or the estimated age of a person’s cardiovascular system given the CVD risk factors, at 13 years. “Each 10 years of excess heart age was associated with 65% increased risk of CVD mortality” [42]. Mexican Americans were next “oldest” at 10.5 years, and Whites were “youngest” at 8.5 years [42]. Cardiovascular health has declined over the last 30 years for all racial and ethnic groups, yet the Black-White CVD -gap is narrowing. [43]. Recent evidence, however, shows that it is not because minority cardiovascular health is improving, but rather because White cardiovascular health is worsening [44].

## 4. Gender Differences across the Life Course

**Figure 4:**
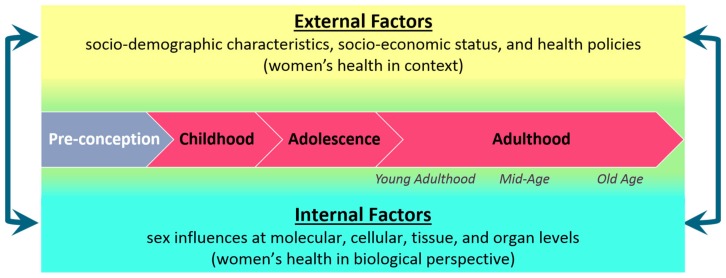
Multidimensional Model of Women’s Health across the Life Course. Internal and external factors, shown here continuously along the top and bottom, both 1. affect the individual (symbolized via flow through the middle) and 2. interact with each other (symbolized via the dual side arrows) across the life course. (This figure has not been referred to within the text of the manuscript, please confirm.)

Women’s health is best understood as a dynamic, multidimensional construct resulting from both internal factors, at the individual-level, as well as external factors, such as sociodemographic characteristics; furthermore, these factors are important to consider across the life course and are subject to change over time as illustrated in Figure 4. To obtain a comprehensive understanding of women’s health, biology at different stages of life must be considered along with cascading and cumulative effects. Distinct diseases and disorders affect women and girls at different ages than men and boys. As explained above, a gendered approach to medicine can be beneficial to adults and should be considered across the life course, including developmental health in children, reproductive and other health contexts in adults, and health concerns affecting older adults [4,45]. To offer some development examples, consider autism spectrum disorder (ASD) and asthma. ASD is a developmental disorder subject to differences by age that displays a striking sex difference; almost five times as many boys are diagnosed with autism than girls. Although there is some evidence for a “female protective effect” [46], the pattern could also reflect a sex bias in diagnosis. Dworzynski and colleagues found that girls are less likely than boys to meet the diagnostic criteria for ASD, even when their ASD-like traits are similar in severity [47]. Furthermore, girls with high-functioning ASD are generally diagnosed later in life than boys. Similarly, asthma is another example with complex sex and age influences. While asthma affects more boys before puberty, it is more prevalent and severe in women in adulthood. This reversal in prevalence may be largely due to the role of sex steroid hormones: estrogens (which regulate the release of proinflammatory cytokines) and testosterone (which suppresses asthma) [48].

Pregnancy is a unique women’s health concern; pregnancy, its complications, and sequelae can also have profound effects on a woman’s subsequent health. For example, pre-eclampsia, which affects 2–8% of pregnancies, puts a woman at risk for later CVD and stroke [49]. In fact, according to Chen and colleagues, “a history of pre-eclampsia increases cardiovascular risk by two to four times, which is comparable with the risk induced by smoking” [50]. Another pregnancy-related condition, gestational diabetes mellitus, increases the risk of developing type 2 diabetes [51,52]. Shifting into later adulthood, while research suggests that stroke was more common in men, the picture seems to be getting more complicated as women are living longer. As would be expected due to age, postmenopausal women show increased stroke rates compared to premenopausal women [53], and while stroke incidence and prevalence rate is still higher in men, the rate of subarachnoid hemorrhage was higher for women. Importantly, due to the differences in age at stroke occurrence, stroke tends to be more severe in women, “with a 1-month case fatality of 24.7% compared with 19.7% for men”. For this same reason, following stroke, quality of life and functional outcomes are poorer in women than in men [24]. Lastly, while some data has suggested a decrease in stroke incidence over time, recent results from a study of 1.3 million Cincinnati, Ohio and Northern Kentucky residents demonstrated that the decreasing trend is primarily driven by a reduction in ischemic stroke shown only in men. The majority of stroke victims in this study were female, and stroke incidence in women did not decrease significantly over the study period [54].

According to recent research released from the Alzheimer’s Association International Conference, factors across a woman’s life course may affect subsequent health later in life [55]. Alzheimer’s disease and other dementias disproportionately affect women with almost two-thirds of Alzheimer’s patients in the U.S. being women [55]. While this difference can in part be attributed to longer life spans for women, there is recent evidence suggesting that pregnancy and reproductive history may play a role in dementia risk. Very recently, Gilsanz and Whitmer have shown that women with three or more children were less likely to develop dementia than women with only one child, even after adjusting for other risk factors such as BMI and stroke history [56]. Fox also found that women who had spent more of their lives pregnant were at lower risk for developing Alzheimer’s later in life [57]. Moreover, biological and genetic variations also impact the risk of developing dementia. Researchers also found that each additional miscarriage led to a 9% increased risk of developing dementia in comparison to women who had not experienced miscarriage. Furthermore, in comparison to women who experience menopause later in life (after age 45), those who experience menopause earlier were at 28% greater dementia risk [56].

## 5. NIH Efforts

ORWH and NIH are keenly aware of these gaps across sex and gender, race and ethnicity, and age, and have introduced efforts in all three areas relevant to the ORWH mission of enhancing women’s health research and promoting career development to “raise the bar”, elevating the standards for the health of women. Predominant among these efforts has been the introduction of the Sex as a Biological Variable (SABV) policy notice, introduced in January 2015. The notice is part of the NIH initiative to enhance reproducibility through rigor and transparency and requires scientists submitting applications for NIH funding to report plans to account for SABV in vertebrate animal and human studies. The SABV policy enjoins applicants to account for SABV in research development, analyses, and reporting [58,59,60]. This policy, along with companion guidance, was published with the expectation that researchers embed SABV in their scientific approaches and will study both sexes unless there is a strong scientific justification not to do so [61]. NIH also developed new instructions for applicants to guide them through the process of adhering to the policy and new review criteria for peer reviewers to help them assess the extent to which applicants sufficiently account for this new requirement (resources for peer reviewers). More recently, NIH held a National Academies Workshop on Improving the Health for U.S. Women in 2016. The workshop discussed multiple themes, including the paucity of research on women’s health and the lack of routine disaggregation of data by sex; but of primary importance was the impact of public policy on health [62]. A call for research to understand the consequences of public policies was issued, particularly for the health of women.

As discussed in the introduction, NIH’s initiative for precision medicine (i.e., the All of Us research program) seeks to develop a “national, large-scale research participant group” of one million or more to realize precision medicine for the individual [63]. Unique to this project, participants share their electronic medical record information voluntarily, and, rather than having a sample of hundreds or thousands focusing on a single disease, the All of Us research program plans to build a database of information on healthy individuals and multiple diseases. The size of the sample alone will allow the project to examine internal factors (e.g., genetics, individual-level factors like sex and gender) and external factors (e.g., environment) on a variety of health outcomes. Additionally, NIH has a trans-NIH strategic plan for women’s health research. The current plan outlines six specific goals and objectives, including enhancing sex differences research for both basic and clinical research, and the translation of sex-specific findings into relevant therapies for women and men. Moreover, the plan includes details about actualizing personalized medicine, developing partnerships domestically and across the globe via existing and new technological innovations, and increasing the presence of women in the health research workforce [64]. As NIH advances science for the health of women, a multidimensional approach will serve as a guiding principle. For example, in FY16, NIH continued a partnership with the Tribal Epidemiology Center, United and Southern Eastern Tribes, Inc. (USET). NIH Tribal Health Research Office funded the “Indian Peer-to-Peer Family Curriculum” project to educate tribal communities on pre- and post-natal care. The project specifically targets women as the gatherer of information on healthcare decisions for the family.

ORWH catalyzes research addressing sex and gender and race and ethnicity across the life course through co-funding collaborations with NIH institutes and centers. Over 10 years ago, NIH took a forward-looking approach to address sex differences by recognizing that there were few funding mechanisms to facilitate sex differences research. ORWH established such a mechanism by offering one-million-dollar Specialized Centers of Research on Sex Differences (SCOR) P50 grants in collaboration with participating NIH Institutes and Centers and the FDA Office of Women’s Health. The ORWH SCOR program funded research centers that integrate basic, preclinical, clinical, and translational research to facilitate innovative, interdisciplinary studies on sex differences relevant to the diseases and conditions that affect women’s health. To date, approximately 300 investigators have been funded by SCOR. SCOR is the only disease-agnostic NIH center grant program and has resulted in compelling scientific advances such as a non-antibiotic treatment for urinary tract infections (UTI) [65]. Women are at a higher risk for UTIs than men, and the use of antibiotics to treat UTIs as well as other conditions is leading to more antibiotic-resistant bacteria [38]. Spaulding and colleagues, funded by the SCOR program, found that they could prevent bacteria from binding to the colon of mice using an orally-administered sugar that binds to the pili in the colon and blocks the bacteria [65]. The prevention of bacterial binding reduced UTIs overall in mouse models, providing promising results.

In the last two years ORWH has issued PA-17-101 to provide one year of supplemental funding to currently funded NIH grantees to address the Health of Women of Understudied, Underrepresented, and Underreported (U3) Populations, also referred to as the U3 program. In FY17, 15 applications were funded, totaling approximately $1.7 million. Of the funded supplements, one particularly prominent example is an award funded to an ongoing randomized control trial studying the effects of docosahexaenoic acid (DHA) on reducing early preterm birth (NICHD PI Carlson, Susan; HD083292). The funded supplement supports community-based participatory research (CBPR) approaches to recruit and retain underrepresented minorities, focusing specifically on pregnant, Hispanic women. Utilizing a CBPR approach will allow researchers to investigate barriers in accessing treatment that may uniquely affect pregnant, Hispanic women as well as determine their perception of participating in the research, which could help future researchers in recruiting and retaining minority participants [66]. A second example supplements a grant examining posttraumatic stress disorder (PTSD) symptoms on chronic pain development after sexual assault to examine ethnic disparities in outcome and treatment (NIAMS PI: McLean, Samuel; AR064700). The supplemental funding is being used to examine differences in outcome trajectories and biobehavioral markers between African and European American survivors via flash survey assessments obtained from smartphone data.

A third example from this program is aimed at increasing health literacy in older, African American women and their caregivers as part of the Center for Translational Neurosciences Grant Award (NIGMS PI Garcia-Rill, Edgar; FWA00001119). Investigators believe that by increasing health literacy, they may be able to decrease the cardiometabolic risk factors for cognitive decline in older, African American women. In Arkansas, where the funding is targeted, there is a particularly low health literacy rate and a high rate of Alzheimer’s disease and other dementias among this population. As discussed in the introduction, ORWH has also created an Inclusion Outreach Toolkit to encourage further engagement, recruitment, and retention of women in clinical research. The toolkit provides strategies for overcoming challenges related to these populations in research, presenting five case studies, one of which details a series of cervical cancer clinical trials headed by Dr. Francisco Garcia at the Arizona Cervical Cancer Prevention Unit. From 2002 to 2012, the unit focused on Hispanic and low-income women in southern Arizona who experience unique barriers to care, such as a lack of understanding of the research on cervical cancer, challenges associated with financial burden, and trust issues with medical staff. This work was also supported by the National Institute on Minority Health and Health Disparities (NIMHHD), which works to promote scientific research on improving minority health and reducing health disparities so that all people in the U.S. can live healthy lives [67].

NIH is also working diligently to raise the bar for the health of women across race, ethnicity, and the life course by promoting women’s career development. By supporting women scientists, including women of color, and encouraging career development, ORWH supports the promotion of a diverse research workforce [68]. Among these efforts is the NIH Working Group on Women in Biomedical Careers (womeninscience.nih.gov). The Working Group works to both promote women into biomedical careers, as well as address barriers to progress, striving to support the retention and sustained advancement of women in medicine and research. The Working Group has several committees, including the Women of Color (WOC) committee which created the Women of Color Research Network (WoCRN) to support the unique challenges faced by women of color working in biomedical careers. The WoCRN exists as a LinkedIn page for those interested in supporting the development of a diverse scientific workforce. By utilizing the social media platform, the WoCRN enables distantly located students, researchers, and policy makers to connect and collaborate, while providing resources regarding NIH grants and processes and advice on career development. One other avenue employed by ORWH is an annual symposium convened in honor of Dr. Vivian Pinn, the first full-time director of ORWH. The purpose of the 2018 symposium was to advance women in science through “Catalytic Connections” (video cast). The theme of the 2017 symposium was women as “makers” of healthy societies (video cast). As part of the 2017 symposium, researcher Jennifer Montez, whose research on mortality rates across the U.S. was discussed earlier, presented her work. Lastly, the NIH Wednesday Afternoon Lecture series (WALS), is the highest profile lecture program at the NIH. Part of the WALS nomination process is outreach to enhance the diversity of both researchers selected and the research presented. For example, the 2016–2017 roster consisted of many women scientists, including Drs. Carla Pugh, Linda Buck, Louise McCullough, and Elizabeth Ofili, among others.

## 6. Conclusions

ORWH is working to put science to work for the health of women by introducing new policy, funding cutting-edge research, and promoting women in biomedical careers. As discussed in this paper, the roles of sex, gender, race, and ethnicity intersect and change across stages of the life course. It is critical that individuals, clinicians, researchers, and policy makers communicate effectively to ensure that the multidimensional aspects of women’s lives are considered appropriately to raise the bar for women’s health. ORWH serves as a focal point for NIH research relevant to the health of women funded by the 27 institutes and centers at NIH and provides resources to stimulate such approaches (nih.gov/women). Without an integrated framework, innovations in research may remain fragmented and of limited value. To effectively tackle the problems that patients, researchers, and clinicians face in today’s world, it is crucial to advance a public health perspective with intersectional and interdisciplinary foundations that integrates the multiple dimensions of the health of women. With such an integrated approach, researchers can begin to close the gap across the racial and ethnic divide as well as develop evidence-based, sex and gender appropriate guidelines to enhance the individual health care needs of all women of all ages.
